# Neither Cholinergic Nor Dopaminergic Enhancement Improve Spatial Working Memory Precision in Humans

**DOI:** 10.3389/fncir.2017.00094

**Published:** 2017-12-05

**Authors:** Adeola N. Harewood Smith, Jnana Aditya Challa, Michael A. Silver

**Affiliations:** ^1^Vision Science Graduate Group, University of California, Berkeley, Berkeley, CA, United States; ^2^Department of Electrical Engineering and Computer Science, University of California, Berkeley, Berkeley, CA, United States; ^3^Helen Wills Neuroscience Institute, University of California, Berkeley, Berkeley, CA, United States; ^4^School of Optometry, University of California, Berkeley, Berkeley, CA, United States

**Keywords:** spatial working memory, dopamine, acetylcholine, spatial resolution, attention

## Abstract

Acetylcholine and dopamine are neurotransmitters that play multiple important roles in perception and cognition. Pharmacological cholinergic enhancement reduces excitatory receptive field size of neurons in marmoset primary visual cortex and sharpens the spatial tuning of visual perception and visual cortical fMRI responses in humans. Moreover, previous studies show that manipulation of cholinergic or dopaminergic signaling alters the spatial tuning of macaque prefrontal cortical neurons during the delay period of a spatial working memory (SWM) task and can improve SWM performance in macaque monkeys and human subjects. Here, we investigated the effects of systemic cholinergic and dopaminergic enhancement on the precision of SWM, as measured behaviorally in human subjects. Cholinergic transmission was increased by oral administration of 5 mg of the cholinesterase inhibitor donepezil, and dopaminergic signaling was enhanced with 100 mg levodopa/10 mg carbidopa. Each neurotransmitter system was separately investigated in double-blind placebo-controlled studies. On each trial of the SWM task, a square was presented for 150 ms at a random location along an invisible circle with a radius of 12 degrees of visual angle, followed by a 900 ms delay period with no stimulus shown on the screen. Then, the square was presented at new location, displaced in either a clockwise (CW) or counterclockwise (CCW) direction along the circle. Subjects used their memory of the location of the original square to report the direction of displacement. SWM precision was defined as the amount of displacement corresponding to 75% correct performance. We observed no significant effect on SWM precision for either donepezil or levodopa/carbidopa. There was also no significant effect on performance on the SWM task (percent correct across all trials) for either donepezil or levodopa/carbidopa. Thus, despite evidence that acetylcholine and dopamine regulate spatial tuning of individual neurons and can improve performance of SWM tasks, pharmacological enhancement of signaling of these neurotransmitters does not substantially affect a behavioral measure of the precision of SWM in humans.

## Introduction

Spatial working memory (SWM) refers to the short-term storage of locations of items not currently present in the environment for immediate use. The limits on working memory can be quantified by measuring capacity (the amount of information that can be remembered) as well as precision (the fidelity with which the memorized information is recalled). In the domain of visual SWM, precision is often quantified as the average distance in the visual field between the encoded location and the location reported during retrieval.

Neural correlates of SWM precision have been described in macaque dorsolateral prefrontal cortex (dlPFC). Here, neurons exhibit sustained spiking activity during a delay period between encoding and retrieval, and the magnitude of this activity varies as a function of the remembered location (Funahashi et al., 1989). The spatial tuning of these neurons is analogous to neuronal receptive field size for visually-evoked responses, but the fact that it is associated with a delay period with no visual stimulation distinguishes this memory-related activity from sensory responses.

We employed a pharmacological approach to explore the relationships between a behavioral measure of the precision of SWM and the spatial tuning of sensory responses and visual perception. Acetylcholine is an endogenous neurotransmitter that increases the spatial resolution of visual representations. Specifically, pharmacologically increasing cholinergic signaling reduces excitatory receptive field size in marmoset V1 neurons (Roberts et al., 2005) and decreases the spatial spread of excitatory fMRI responses to visual stimulation in human early visual cortex (Silver et al., 2008). In addition, cholinergic enhancement with the cholinesterase inhibitor donepezil causes changes in visual perception that are consistent with a reduction in excitatory receptive field size (Kosovicheva et al., 2012; Gratton et al., 2017). Moreover, administration of acetylcholine receptor agonists improves spatial tuning of delay period activity in dlPFC neurons and performance on a SWM task in macaque monkeys (Yang et al., 2013; Sun et al., 2017).

Dopamine is another neurotransmitter that has been implicated in regulation of tuning of spatial representations in the brain and SWM. In particular, local administration of drugs that act at D1 dopamine receptors can sharpen the spatial tuning of delay period activity in dlPFC neurons in macaque monkeys performing a SWM task (Williams and Goldman-Rakic, 1995; Vijayraghavan et al., 2007), and some studies have reported improved performance on SWM tasks in humans following administration of dopamine receptor agonists (Luciana et al., 1992; Luciana and Collins, 1997; Müller et al., 1998).

Given these enhancing effects of cholinergic and dopaminergic drugs on spatial representations in visual cortex, visual perception, and working memory, here we asked whether systemically increasing cholinergic transmission with donepezil and dopaminergic transmission with the dopamine metabolic precursor levodopa improves the spatial precision of working memory representations, as measured behaviorally in healthy human subjects.

## Materials and Methods

### Participants

The Committee for the Protection of Human Subjects at the University of California, Berkeley, approved all experimental procedures, and all participants provided written informed consent in accordance with the Declaration of Helsinki before the study began. All subjects reported normal visual acuity, either with or without optical correction. Nineteen participants (4 males and 15 females) completed the donepezil study, and 20 (6 males and 14 females) completed the levodopa/carbidopa study. One female subject from the donepezil study and two female subjects from the levodopa/carbidopa study were excluded from the analyses because their calculated SWM thresholds were greater than the maximum displacement we tested (described in “Stimuli and Task” section below).

Subjects were not enrolled in the study if they reported that they smoked tobacco, were taking any drugs that could affect cholinergic (for the donepezil study) or dopaminergic (for the levodopa/carbidopa study) function, or had a history of substance abuse, heart arrhythmia or heart problems, neurological or psychiatric illness, or liver disease. Because levodopa/carbidopa can cause hypotension, blood pressure was measured just before administration of levodopa/carbidopa (or placebo). Participants were required to have a resting blood pressure reading between 100/60 mmHg and 140/90 mmHg and a pulse rate above 60 bpm to continue in the experiment. Participants’ ages ranged from 18 to 27 (donepezil study) and from 19 to 31 (levodopa/carbidopa study).

### Pharmacology

We employed a double blind within-subject experimental design in which each subject ingested either placebo or an active drug (5 mg donepezil for the acetylcholine study; 100 mg levodopa/10 mg carbidopa for the dopamine study) on different days. Carbidopa was co-administered in order to inhibit peripheral metabolism of levodopa, thereby allowing more levodopa to cross the blood-brain barrier (Olanow et al., 2000). There were three experimental sessions per subject. For the initial baseline session, subjects were acclimated to the SWM task, and no pill was administered. Data from the baseline session were used to optimize the stimuli for each subject in the subsequent pharmacological sessions.

At the beginning of the second session, subjects ingested either a drug or placebo pill, and at the beginning of the third session, subjects ingested whichever pill (drug or placebo) they did not take during the second session. Participants waited 3 h after ingesting donepezil and 45 min after ingesting levodopa/carbidopa to begin the SWM task, intervals that correspond to the time to reach peak plasma concentration after oral ingestion for each drug (donepezil: Rogers and Friedhoff, 1998; levodopa/carbidopa: Olanow et al., 2000). The third session occurred at least 2 weeks after the second session to allow the drug to be completely eliminated from the body before further testing. The half-life of donepezil is 80 h (Rogers and Friedhoff, 1998), and the half-life of levodopa/carbidopa is 1–2 h (Olanow et al., 2000; Nyholm et al., 2012). The order of drug/placebo administration in the two sessions was counterbalanced for each of the two studies (acetylcholine and dopamine).

### Stimuli and Task

**Figure 1:**
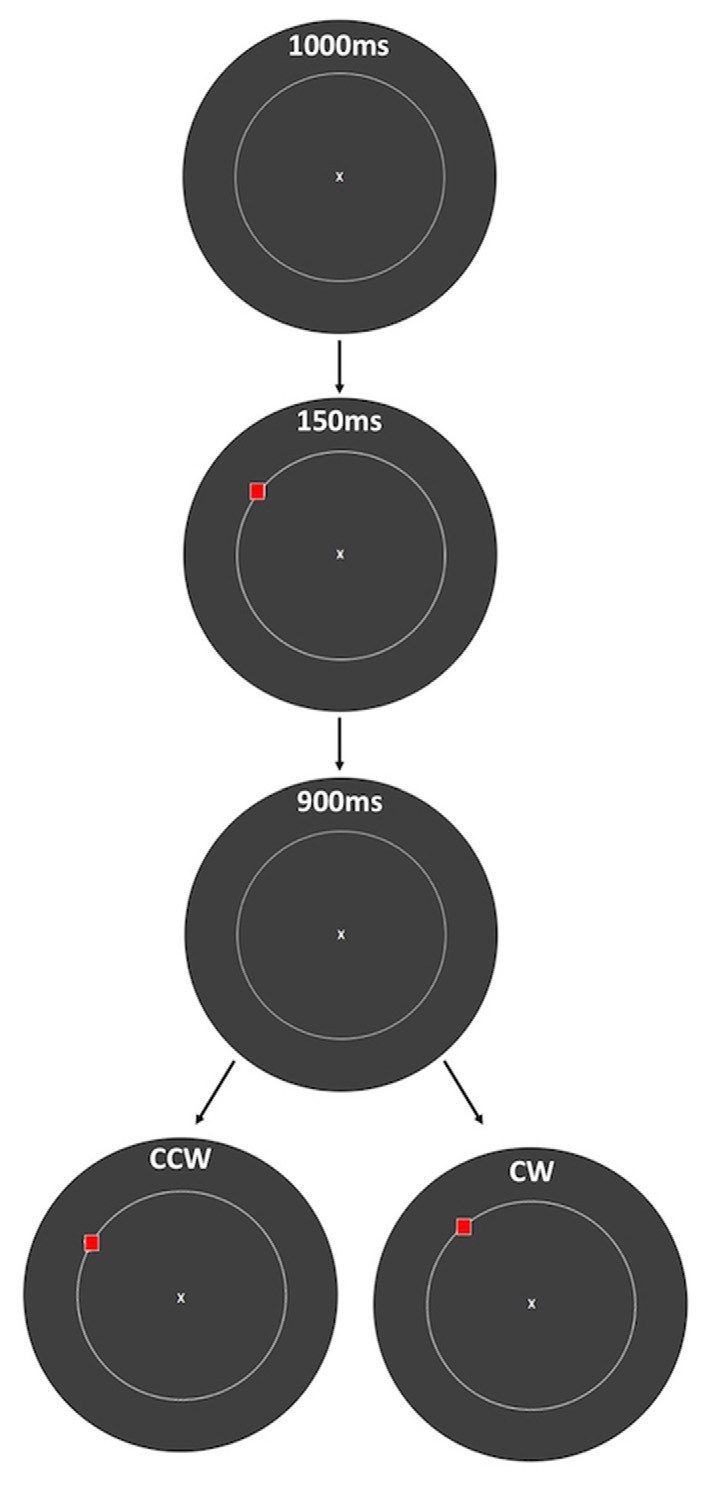
Spatial working memory (SWM) task. At the beginning of each trial, subjects viewed a fixation point for 1 s. A red square was then presented for 150 ms, followed by 900 ms of a blank screen and then presentation of the same red square, displaced either clockwise (CW) or counterclockwise (CCW) from its original location along a circle. Auditory feedback (150 ms) was given immediately after the response was made, followed by the beginning of the next trial. The amount of displacement was defined as the polar angle between the two red squares (10 degrees in this example), and subjects indicated the direction of displacement with a key press. The circle is displayed in this figure to indicate the set of possible stimulus locations, but it was not visible to the subjects.

Each trial began with a 1000 ms period of central fixation on a 1 × 1 degree white “X” at the center of the screen, followed by 150 ms presentation of the stimulus to be encoded: a 1 × 1 degree red square presented 12 degrees of visual angle from fixation (Figure 1). Following a 900 ms delay period, the stimulus was displaced from its randomly selected original location, in either a clockwise (CW) or counterclockwise (CCW) direction along the circle. This probe stimulus remained on the screen until the subject made a response. Subjects responded by pressing the “1” key on a keypad for CCW and “2” for CW displacement, and auditory feedback was provided to indicate whether the response was correct or incorrect, followed immediately by the beginning of the 1000 ms fixation period of the next trial.

Testing was conducted in a light attenuated room. Stimuli were presented on a NEC Multisync FE992 CRT monitor with a screen resolution of 1280 by 1024 and a refresh rate of 75 Hz using Psychopy software (Peirce, 2009). Subjects viewed the monitor from a distance of 50 cm, and a chin and forehead rest kept the head position stabilized.

There were 120 possible locations for the stimulus to be remembered, all of which were on an invisible circle with a 12-degree radius. A circular aperture was attached to the front of the screen so that subjects could not use the corners or edges of the monitor frame as spatial cues during the SWM task. Subjects were instructed to maintain central fixation throughout the trial, and the experimenter monitored their eye position with an infrared camera. If fixation was not maintained during the trial, the experimenter reminded the subject to maintain fixation, and that trial was excluded from analysis and not repeated. The 1000 ms fixation period for the next trial then began. On average, 0.29% of trials were excluded due to failure to maintain fixation.

We conducted a control experiment to determine the size of the window for which the two experimenters who conducted the SWM experiments were able to reliably detect eye movements. In this control experiment, the subject fixated for 1 s, and then a 0.5 degree diameter circle was presented at 0.5, 1, 1.5, 2, or 2.5 degrees eccentricity from fixation for 500 ms. For half of the trials, the circle was red, indicating to the subject that he or she should make an eye movement to the stimulus location and then immediately back to the fixation point. For the remaining trials, the stimulus was blue, indicating that the subject should maintain central fixation. The experimenter then reported whether an eye movement had occurred or not, based on the infrared video of the subject’s eye. At each eccentricity, there were 120 possible stimulus locations that comprised an invisible circle. Psychometric functions of percent correct trials vs. stimulus eccentricity were computed, and Weibull functions were fit to these functions to determine the eccentricity corresponding to 75% correct performance (2.1 degrees of visual angle for experimenter 1 and 1.6 degrees for experimenter 2). Across all eccentricities, the mean hit rate was 61%, and the mean correct reject rate was 75%. It should be noted that we used a 500 ms stimulus presentation time in this control experiment instead of the 150 ms stimulus duration used in the SWM experiments, as 150 ms is not enough time for the subjects to make an eye movement to the target while it was still being displayed. This 150 ms stimulus duration was selected to discourage eye movements to the stimulus to be remembered during the SWM task.

During the SWM experiment, participants were encouraged to take breaks whenever they wanted to, and they communicated this by either withholding their response or informing the experimenter, who would then pause the experiment after the subject’s response. Additionally, the experimenter explicitly asked participants if they wanted to take a break every time they completed 20% of the trials (total of four times).

**Figure 2:**
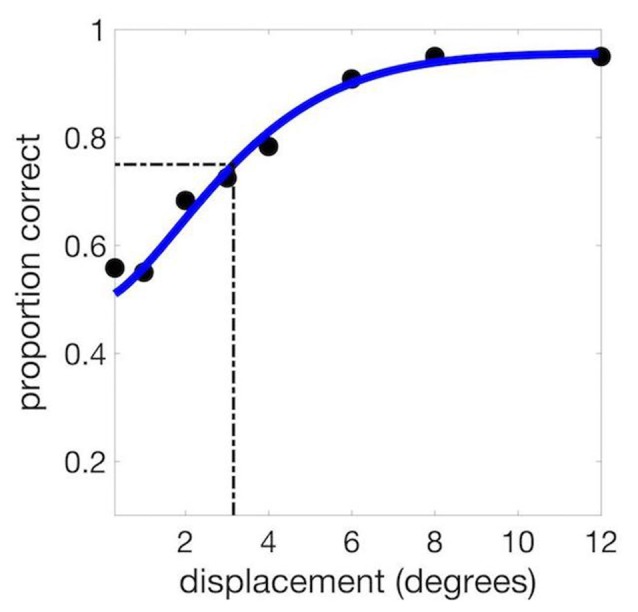
Example psychometric curve from a single experimental session. We used the psychometric function to calculate the threshold at 75% correct (3.15 degrees in this example).

For the baseline session, the set of displacements was 0.3, 1, 2, 3, 4, 6, 8 and 10 degrees (defined as the polar angle between the encoded stimulus and the probe). Performance was plotted as a function of this displacement angle (Figure 2), and the threshold from the resulting psychometric function was defined as the displacement corresponding to 75% correct for the fitted function. We used Palamedes Toolbox for Matlab (Prins and Kingdom, 2009) to compute values for the free parameters of alpha (threshold), beta (slope) and lambda (lapse rate, or the proportion of incorrect responses for trials with very large displacements, bounded at 0 and 1).

For the pharmacology sessions, displacements ranged from 0.3 to 12 degrees of polar angle, with the intervening displacements at 10%, 30%, and 60% above and below the subject’s threshold (computed from the baseline session). The baseline session had 960 trials, and the pharmacology sessions had 1080 trials each. Due to experimenter error, for a subset of the participants (10 in the donepezil study and seven in the levodopa/carbidopa study), data were not collected at a displacement of 60% above threshold. In order to estimate the effect of this missing data, we removed the 60% above threshold data point from those subjects with a complete data set and then recomputed the thresholds. We found that there was no significant difference between thresholds calculated from the complete data set and those from the data that were missing the 60% above threshold value (*t*_(37)_ = −1.49, *p* = 0.14). We therefore included all collected data in our analyses.

## Results

**Figure 3:**
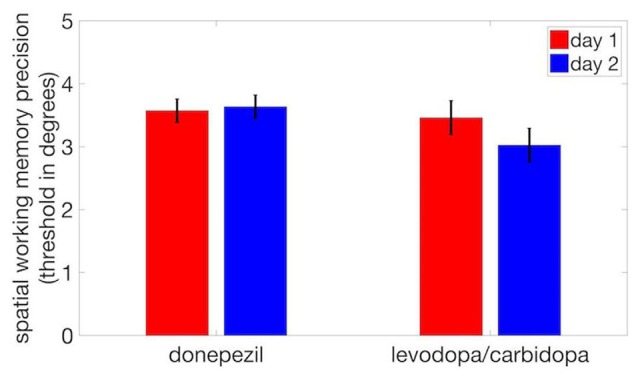
No evidence of practice effects on SWM precision. We observed no significant difference in thresholds between day 1 and day 2. Error bars are within-subject standard errors of the mean (SEM).

To assess stability of SWM precision across multiple testing sessions, we compared threshold displacement (measured in units of degrees of polar angle) for the two pharmacology sessions in each study (acetylcholine and dopamine) using paired *t*-tests. Half of the subjects in each study received the drug in the first session and placebo in the second, and the other half were administered placebo in the first session and the active drug in the second. We found no significant difference in threshold between Day 1 and Day 2 for either donepezil (*t*_(17)_ = 0.10, *p* = 0.73) or levodopa/carbidopa (*t*_(17)_ = 0.49, *p* = 0.12; Figure 3), indicating that performance was stable and that no measurable learning occurred between the first and second pharmacology sessions.

**Figure 4:**
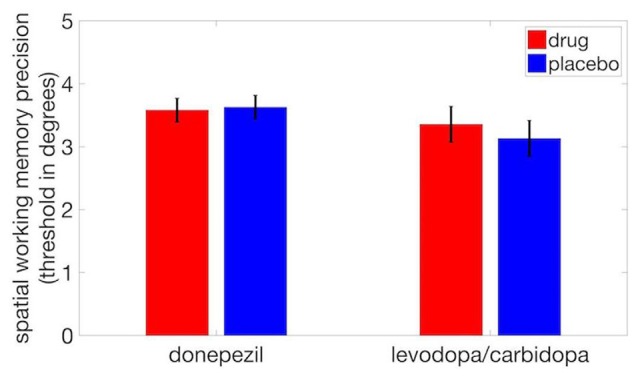
Neither donepezil nor levodopa/carbidopa significantly affected displacement threshold on the SWM task. Error bars are within-subject SEM.

We observed no significant difference in SWM precision thresholds between donepezil and placebo (*t*_(17)_ = −0.25, *p* = 0.81) or between levodopa/carbidopa and placebo (*t*_(17)_ = 0.80, *p* = 0.44; Figure 4). Thus, even though acetylcholine regulates neuronal receptive field size, perceptual measures of spatial tuning, and the spatial tuning of mnemonic responses in dlPFC, cholinergic enhancement with donepezil had no detectable effect on the precision of SWM. Similarly, although local administration of dopaminergic drugs modulates the spatial tuning of dlPFC neurons during performance of a SWM task, we found that systemic administration of levodopa/carbidopa did not significantly alter a behavioral measure of SWM precision.

**Figure 5:**
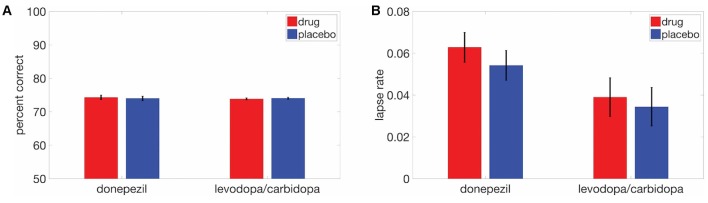
Neither donepezil nor levodopa/carbidopa significantly affected **(A)** overall performance or **(B)** lapse rate. Error bars are within-subject SEM.

We also examined the effects of cholinergic and dopaminergic enhancement on overall task performance (percent correct) and again observed no significant drug effects (donepezil: *t*_(17)_ = 0.46, *p* = 0.65; levodopa/carbidopa: *t*_(17)_ = 0.50, *p* = 0.62; Figure 5A). The absence of drug effects was not due to ceiling effects on performance. Average percent correct values and standard deviations across all displacements in the donepezil study were 73.3 ± 3.0% in the placebo condition and 74.0 ± 3.0% in the donepezil condition. In the levodopa/carbidopa study, these values were 73.9 ± 3.3% for placebo and 74.0 ± 2.5% for levodopa/carbidopa. In addition, across both studies, mean overall performance ranged from approximately chance levels at the smallest displacement (53% at 0.3 degrees) to nearly perfect at the largest displacement (95% at 12 degrees), indicating that the range of displacements we used was large enough to accurately measure SWM precision.

Finally, there were no detectable effects of either donepezil (*t*_(17)_ = 1.21, *p* = 0.24) or levodopa/carbidopa (*t*_(17)_ = 0.50, *p* = 0.62) on lapse rate (Figure 5B), a parameter of the fitted psychometric function that corresponds to the proportion of trials for which subjects responded incorrectly at the highest displacements.

Both cholinergic and dopaminergic drugs can exhibit inverted-U-shaped dose-response functions (reviewed in Bentley et al., 2011 for acetylcholine and Cools and D’Esposito, 2011 for dopamine). In addition, baseline performance on a working memory task has been shown to predict whether systemic administration of a dopaminergic drug enhances or impairs performance relative to this baseline (Kimberg et al., 1997; Kimberg and D’Esposito, 2003). Moreover, individual differences in striatal dopamine synthesis capacity are correlated with working memory capacity (Cools et al., 2008), and individual differences in accuracy on a working memory task are predicted by a polymorphism in the dopamine beta-hydroxylase gene (Parasuraman et al., 2005), which codes for an enzyme that metabolizes dopamine. These findings raise the possibility that individual differences in SWM precision at baseline may reflect differences in cholinergic and/or dopaminergic tone that could influence drug effects on SWM precision.

**Figure 6:**
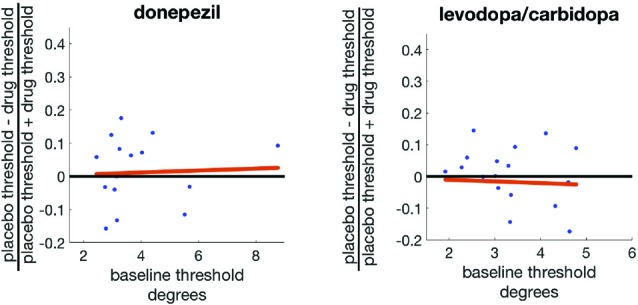
Baseline SWM precision does not predict the effects of either donepezil or levodopa/carbidopa on SWM precision.

We therefore correlated the baseline threshold for each subject with a contrast index ((SWM placebo threshold − SWM drug threshold)/(SWM placebo threshold + SWM drug threshold)) for each study. This contrast index will have a value of zero when the drug has no effect on displacement threshold, positive values when the drug enhances precision (decreases threshold), and negative values when the drug reduces precision (increases threshold). This correlation was not significant for either donepezil (*r* = 0.19, *p* = 0.45) or levodopa/carbidopa (*r* = −0.06, *p* = 0.81; Figure 6).

**Figure 7:**
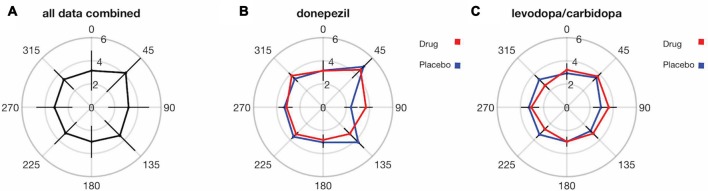
SWM precision does not significantly vary across the visual field, and there were no detectable effects of either donepezil or levodopa/carbidopa on SWM precision at any visual field location. Distance from center indicates the SWM threshold in units of degrees for each visual field location. Error bars are standard deviations in **(A)** and within-subject SEM in **(B,C)**.

Finally, we explored whether SWM precision varies across different locations in the visual field. There is a well-established lower visual field advantage in performance for a variety of visual perception tasks (He et al., 1996; Rubin et al., 1996; Abrams et al., 2012; Fortenbaugh et al., 2015). We therefore plotted SWM precision as a function of visual field location (based on the stimulus to be encoded), binned into eight regions, each comprising 45 degrees of polar angle (Figure 7A). Data from placebo and drug sessions were combined for these analyses. Overall, there were no significant differences between SWM precision in the upper and lower halves of the visual field (*t*_(35)_ = −0.70, *p* = 0.48) or between the left and right hemifields (*t*_(35)_ = −1.25, *p* = 0.21). Lower visual field advantages in perception have often been measured for stimuli on or near the vertical meridian (He et al., 1996; Fortenbaugh et al., 2015). We therefore compared SWM precision in the upper and lower visual fields using only trials with stimulus locations within 22.5 degrees of the vertical meridian and again found no significant difference (*t*_(35)_ = 0.71, *p* = 0.47).

The oblique effect is another well-studied anisotropy in visual perception across visual field locations (Appelle, 1972; Rokem and Silver, 2009), characterized by enhanced perception along the cardinal compared to the oblique axes of the visual field. We therefore tested for an oblique effect in SWM precision. We observed significantly greater SWM precision for locations near (within 22.5 degrees of polar angle) the cardinal compared to the oblique axes (*t*_(35)_ = 2.24, *p* = 0.03; Figure 7A). However, there were no significant differences in the magnitude of the drug effect (placebo SWM threshold—drug SWM threshold) between the oblique and the cardinal axes for either donepezil (*t*_(17)_ = −1.21, *p* = 0.23; Figure 7B) or levodopa/carbidopa (*t*_(17)_ = 1.52, *p* = 0.15; Figure 7C).

## Discussion

The purpose of this study was to investigate the effects of cholinergic and dopaminergic enhancement on SWM precision, using the cholinesterase inhibitor donepezil and the dopamine metabolic precursor levodopa, respectively. We found no detectable effects of enhanced acetylcholine and dopamine signaling on either SWM precision or task performance.

### Acetylcholine

At the single neuron level, local administration of acetylcholine reduces excitatory receptive field size in marmoset V1 (Roberts et al., 2005), thereby enhancing the spatial resolution of visually-evoked responses. At the population level, reduced receptive field size corresponds to decreased spatial extent of excitatory visual responses in retinotopic visual cortical areas, and this is what was found for fMRI responses in early visual cortex following systemic administration of donepezil to healthy human subjects (Silver et al., 2008).

At the perceptual level, systemic administration of donepezil reduces orientation-selective surround suppression (Kosovicheva et al., 2012). Specifically, donepezil diminished the impairment of contrast discrimination within a target grating due to presentation of a high-contrast surrounding grating. This cholinergic effect on surround suppression was specific to the condition in which the target grating and surround shared the same stimulus orientation, implicating early visual cortical circuits that exhibit orientation-selective surround suppression (Blakemore and Tobin, 1972; Cavanaugh et al., 2002).

Systemic administration of donepezil also has been shown to enhance contrast discrimination of a target with flankers, but only for intermediate target-flanker distances (Gratton et al., 2017). Modeling of facilitatory and suppressive effects of the flankers indicated that donepezil improved performance by reducing the spatial extent of facilitatory target/flanker interactions, consistent with reduced excitatory receptive field size. Thus, converging lines of evidence demonstrate that acetylcholine enhances spatial precision of both visual cortical neuronal representations and visual perception.

Acetylcholine has also been examined in SWM tasks. Lesions of cholinergic inputs to macaque dlPFC selectively impaired SWM performance but did not affect performance of decision-making and episodic memory tasks (Croxson et al., 2011). Local administration of nicotinic acetylcholine receptor agonists in macaque dlPFC increased delay period activity in a SWM task for the neuron’s preferred location but not the nonpreferred location, thereby improving spatial tuning of memory-related activity (Yang et al., 2013; Sun et al., 2017). Moreover, systemic administration of the α7-nicotinic acetylcholine receptor agonist PHA543613 can improve SWM task performance in macaque monkeys (Yang et al., 2013), although precision of SWM was not measured in this study. However, systemic cholinergic enhancement with the cholinesterase inhibitor physostigmine improved accuracy in a spatial attention but not a SWM task in human subjects (Bentley et al., 2004).

### Dopamine

Many studies have shown that pharmacological manipulation of dopaminergic signaling through iontophoresis of dopaminergic drugs in macaque dlPFC enhances spatial tuning of delay-period activity while monkeys are performing a SWM task (Williams and Goldman-Rakic, 1995; Vijayraghavan et al., 2007). There is also some evidence that systemic administration of dopamine receptor agonists can improve performance on a SWM task in human subjects. Systemic administration of the D2/D1 receptor agonist bromocriptine was reported to enhance SWM but not object working memory performance (Luciana et al., 1992; Luciana and Collins, 1997), but other studies found no effect of systemic administration of bromocriptine on behavioral measures of SWM (Kimberg et al., 1997; Müller et al., 1998; although Müller et al., 1998 reported improved SWM performance following systemic administration of the D1/D2 receptor agonist pergolide). Our study differed from those summarized here in that these studies measured overall accuracy or performance on a SWM task. To our knowledge, our study is the first to examine the effects of dopaminergic enhancement on a behavioral measure of SWM precision.

### Methodological Considerations

For our study, we selected drugs that enhance cholinergic and dopaminergic function in a manner that is highly physiologically relevant to endogenous neurotransmitter signaling. Donepezil enhances cholinergic transmission by blocking the enzyme that inactivates acetylcholine after it has been released into the synaptic cleft, thereby prolonging the effective lifetime of acetylcholine in the synapse. Levodopa is metabolically converted to dopamine through the biochemical mechanisms that generate endogenous dopamine. The actions of these drugs are therefore distinct from those of receptor agonists and antagonists that bind directly to neurotransmitter receptors and alter activity in a manner that is largely independent of ongoing endogenous neurotransmitter signaling.

Although the use of drugs that modulate endogenous signaling has the benefit of physiological relevance, it is possible that more selective pharmacological manipulations that target particular receptor subtypes (like those typically used in single-unit studies of memory-related activity in macaque dlPFC neurons) could reveal cholinergic and/or dopaminergic effects on a behavioral measure of SWM precision in humans.

The acute dose of donepezil that we used was 5 mg, corresponding to the lowest dose prescribed clinically for daily administration. While it is possible that cholinergic effects on SWM precision would be observed at higher doses of donepezil, previous studies in our lab have documented statistically significant effects of a single dose of 5 mg donepezil on spatial extent of fMRI responses in visual cortex (Silver et al., 2008), the effects of endogenous spatial attention on visual perception (Rokem et al., 2010), a behavioral measure of surround suppression (Kosovicheva et al., 2012), and the spatial extent of facilitatory target/flanker interactions in visual perception (Gratton et al., 2017). Similarly, the dose of levodopa/carbidopa that we employed was 100 mg/10 mg, and 100 mg levodopa has been shown to have significant effects on fMRI responses in the striatum to stimuli associated with punishment (Wittmann and D’Esposito, 2015), functional connectivity of fMRI signals (Kelly et al., 2009), and the magnitude of striatal reward prediction errors (Pessiglione et al., 2006).

Many cholinergic and dopaminergic drugs can produce an inverted-U-shaped dose-response function, in which a small increase in signaling can benefit task performance and increase regional brain activity, but a larger increase can cause effects in the opposite direction (reviewed in Bentley et al., 2011; Cools and D’Esposito, 2011). An inverted-U-shaped profile has also been reported for cholinergic (Yang et al., 2013) and dopaminergic (Vijayraghavan et al., 2007) effects on spatial tuning of dlPFC neuronal delay period responses. While it is possible that a different dose of donepezil or levodopa/carbidopa in our study could have produced different results, we found no significant correlation between a subject’s baseline SWM precision and effects of donepezil or levodopa/carbidopa on SWM precision for that subject. This lack of correlation could indicate that individual differences in baseline cholinergic or dopaminergic tone do not predict drug effects on SWM precision. However, it is also possible that SWM precision may not be an accurate proxy for baseline cholinergic or dopaminergic tone.

It is also possible that larger sample sizes would have revealed effects of dopaminergic and/or cholinergic enhancement on SWM precision. Our analysis included complete data sets from 18 participants in each study, a sample size that is comparable to previous studies that have documented significant effects of cholinergic enhancement on perception and dopaminergic enhancement on working memory (cholinergic studies: Kosovicheva et al., 2012, 19 subjects; Gratton et al., 2017, 28 subjects; Rokem et al., 2010, 20 subjects; Bentley et al., 2004, 18 subjects; dopaminergic studies: Kimberg et al., 1997, 31 subjects; Luciana et al., 1992, 8 subjects; Müller et al., 1998, 32 subjects). We also note that our subject pool differed from that of most other studies in gender balance, as 14/18 of our subjects in the donepezil study and 12/18 in the levodopa/carbidopa study were female.

Given the observed variance in our measurements and our sample sizes, the within-subject SWM threshold difference between the placebo and drug conditions would have needed to be 0.39 degrees (10.8% change from placebo) in the donepezil study and 0.60 degrees (19.1% change from placebo) in the levodopa/carbidopa study in order to produce a significant drug effect at *p* = 0.05. By comparison, the spatial spread of the excitatory fMRI response to visual stimulation was reduced by 8.5% in area V1 when subjects received donepezil compared to placebo (Silver et al., 2008). Moreover, local administration of acetylcholine reduced receptive field length of V1 neurons by 15.3% (Roberts et al., 2005). In our study, percent change in SWM threshold was 1.3% (donepezil threshold numerically less than placebo) and 7.2% (levodopa/carbidopa threshold numerically greater than placebo).

Another consideration is that we used a delay period of 900 ms without a visual mask. In principle, persistence of the sensory response to the stimulus to be remembered could have aided subjects’ performance on the SWM task. However, this type of retinal persistence, often studied in the psychological literature as iconic memory, fades after 300 ms (Sperling, 1960), an interval much shorter than our 900 ms delay period. In addition, our use of a delay period of 900 ms with no mask is consistent with several previous studies of visual and visuospatial working memory (Alvarez and Cavanagh, 2004; Vogel and Machizawa, 2004; Bo and Seidler, 2009).

Much of the evidence for cholinergic and dopaminergic effects on spatial tuning of working memory representations comes from studies of macaque dlPFC neurons. Although the SWM task we employed is very similar to that used in the macaque studies, recent evidence from human patients with lesions to dlPFC has raised questions about the homologies between humans and macaques in this region (Mackey et al., 2016). Specifically, patients with dlPFC lesions had normal accuracy on a SWM task, while patients with precentral sulcus lesions had lower accuracy when making saccades to a remembered location. However, these inaccurate saccades were typically followed by corrective saccades, indicating that the deficit in patients with precentral sulcus lesions may be in the domain of executive function rather than reduced precision of SWM representations.

These lesion results are supported by a recent transcranial magnetic stimulation study in which disruption of human dlPFC did not affect accuracy of memory-guided saccades (Mackey and Curtis, 2017). However, disruption of topographically-organized precentral sulcus and intraparietal sulcus regions impaired SWM accuracy (Mackey and Curtis, 2017) in a way that is consistent with analogous studies in macaque frontal eye fields (FEF) and lateral intraparietal area (LIP). Taken together, the data suggest that the dlPFC circuits that subserve SWM in macaque monkeys may not have a direct homolog in human dlPFC but that other frontal and parietal regions that support SWM may be more homologous in the two species. These species differences in the functional networks underlying SWM may be accompanied by neurochemical differences as well, possibly accounting for the fact that both acetylcholine and dopamine have well documented effects on neural correlates of SWM in the macaque dlPFC but no observable effect on behavioral SWM precision in humans. An important direction for future research is to characterize cholinergic and dopaminergic effects on neural correlates of SWM in those frontal and parietal regions that appear to have functional homologies in humans and macaques.

### Differences between Spatial Precision of Perception and Working Memory Representations

Our results support a distinction between the limits of spatial resolution in visual cortical neurons and visual perception and the corresponding limits in SWM representations. Although there are clear cholinergic effects on spatial resolution at the level of single neurons (Roberts et al., 2005), fMRI responses (Silver et al., 2008), and visual perception (Gratton et al., 2017), we found no evidence for cholinergic effects on the precision of SWM, as measured behaviorally in human subjects.

We also found no evidence for visual field asymmetries in the precision of SWM, a result that also indicates fundamental differences between the spatial resolution of perception and memory. Previous studies have documented a clear lower visual field advantage in visual crowding tasks (He et al., 1996; Fortenbaugh et al., 2015). Visual crowding refers to the reduction in discriminability of a stimulus in the peripheral visual field when it is flanked by other stimuli. The strength of crowding depends strongly on the distance between the target and flankers, and the minimal target/flanker distance that enables a certain level of performance is known as the critical spacing, which is a measure of spatial resolution of visual perception. We have recently shown that critical spacing is smaller in the lower compared to the upper visual field** (**Harewood et al., 2016). In the present study, this upper/lower visual field difference was not observed for SWM precision, a measure of the spatial resolution of working memory representations.

Thus, even though SWM representations must be derived from perceptual representations to some extent, we have found fundamental dissociations between the spatial resolution of SWM and perception, both in their associated neurochemical mechanisms as well as visual field anisotropies.

## Author Contributions

ANHS, JAC and MAS designed the experiment and gave final approval of this version of the manuscript to be published. ANHS and JAC collected data and created figures for the article. ANHS and MAS created data analysis procedures, interpreted data, and drafted and edited the manuscript.

## Conflict of Interest Statement

The authors declare that the research was conducted in the absence of any commercial or financial relationships that could be construed as a potential conflict of interest. The handling editor is currently co-organizing a Research Topic with one of the reviewers AD, and confirms the absence of any other collaboration.
